# Coronavirus disease 2019 and radiation oncology—survey on the impact of the severe acute respiratory syndrome coronavirus 2 pandemic on health care professionals in radiation oncology

**DOI:** 10.1007/s00066-022-01903-8

**Published:** 2022-02-23

**Authors:** Marco M. E. Vogel, Carmen Kessel, Kerstin A. Eitz, Stephanie E. Combs

**Affiliations:** 1 Department of Radiation Oncology, University Hospital Klinikum rechts der Isar, Technical University of Munich (TUM), Ismaninger Straße 22, 81675 Munich, Germany; 2 Institute for Radiation Medicine (IRM), Department of Radiation Sciences (DRS), Helmholtz Zentrum München, Neuherberg, Germany; 3 Comprehensive Cancer Center Munich of the Technical University of Munich (CCCM TUM), Munich, Germany; 4 Partner Site Munich, Deutsches Konsortium für Translationale Krebsforschung (DKTK), Munich, Germany

**Keywords:** Mental health support, Child care programs, Coronavirus disease 2019, Severe acute respiratory syndrome coronavirus 2, Protective clothing

## Abstract

**Background:**

The severe acute respiratory syndrome coronavirus 2 (SARS-CoV-2) pandemic has changed the lives of most humans worldwide. The aim of this study was to evaluate the impact of the SARS-CoV‑2 pandemic on health care professionals (HCPs) in radiation oncology facilities.

**Methods:**

We distributed an online survey to HCPs in radiation oncology (physicians, medical physics experts, radiology assistants/radiation therapists, nurses, and administrative personnel). The survey was completed by 334 participants between May 23 and June 9, 2020.

**Results:**

In 66.2% of the cases, HCPs reported a shortage of protective clothing. The protective measures were regarded as very reasonable by 47.4%, while 0.8% regarded them as not reasonable (rather reasonable: 44.0%; less reasonable 7.8%). 29.0% of the participants had children who needed care. The most frequently used care options were public emergency childcare (36.1%) and private childcare (e.g. relatives/friends). HCPs reported about additional work burden (fully agreed: 27.2%, rather agreed: 34.4%, less agreed: 28.2%, not agreed: 10.2%), and reduced work satisfaction (fully agreed: 11.7%, rather agreed: 29.6%, less agreed: 39.8%, not agreed: 18.9%). 12.9% and 29.0% of the participants were fully or rather mentally strained (less mentally strained: 44.0%, not mentally strained: 14.1%).

**Conclusion:**

We must learn from this pandemic how to prepare for further outbreaks and similar conditions. This includes the vast availability of protective clothing and efficient tracing of infection chains among the HCPs, but also secured childcare programs and experienced mental health support are crucial. Further, work satisfaction and appreciation by employers is essential.

**Supplementary Information:**

The online version of this article (10.1007/s00066-022-01903-8) contains supplementary material, which is available to authorized users.

## Introduction

With the outbreak of the coronavirus disease 2019 (COVID-19) caused by the severe acute respiratory syndrome coronavirus 2 (SARS-CoV-2) [1] in December 2019 in Wuhan, China [2] the medical community has been faced with new challenges. The rapidly increasing numbers of infections all over the world posed immense challenges to the health care system. Especially the medical disciplines dealing with vulnerable cancer patients face increasing pressure and responsibility in taking care of their patients in this crisis [3]. On the one hand, intensive care capacity must be preserved for the treatment of SARS-CoV‑2 patients; on the other hand, cancer requires fast and experienced treatment which in most cases should not be postponed. Data from several trials have shown that prolongation of treatment initiation in cancer patients such as head-and-neck tumours, breast cancer or tumours of the central nervous system lead to significantly decreased survival rates [4–6]. Exemplarily, a delay between surgery and adjuvant radiotherapy beyond 7 weeks in patients with head-and-neck cancer is associated with decrements in overall survival [7]. In spite of several warnings of a forthcoming pandemic, health care professionals (HCPs) were overwhelmed by the new situation and recommendations for oncologic care were desperately needed: Very quickly several groups adapted their guidelines and sought to distribute them rapidly. We previously reported our first statement on preparation for the COVID-19 pandemic in large German-speaking university-based radiation oncology departments [8]. Further, in a consensus statement, we described recommendations for the management of high-grade gliomas during the COVID-19 pandemic [9]. Adaption of treatments, hypofractionation and modification of chemotherapy to reduce immunosuppression were discussed controversially, but depending on the countries, institutions and other very specific individual factors, all of those might be feasible in such times [9, 10]. Matuschek et al. previously showed a significant impact of SARS-CoV‑2 on the treatment regimens and work routine for radiation oncologists in Germany, Austria, and Switzerland in their survey [11].

Radiation oncology is an integral part of cancer treatment, and the care of oncologic patients in this discipline needs—besides extensive knowledge and technology—sensitivity and empathy. It is known that HCPs in oncology are exposed to high levels of emotional pressure, and specific counselling can be helpful in coping with the very complex oncological situations. The SARS-CoV‑2 pandemic certainly has an additional significant impact on the well-being of HCPs in radiation oncology and thus might affect the daily work and private life. In this present survey, we aimed at evaluating the impact of the SARS-CoV‑2 pandemic on HCPs in radiation oncology facilities with particular focus on concerns and needs in personal and professional life during the pandemic.

## Materials and methods

A team of experienced radiation oncologists developed a questionnaire with 42 items on the impact of the SARS-CoV‑2 pandemic on HCPs in radiation oncology facilities. Since this was an exploratory survey, the questions were specifically developed for the purpose of this study. Questions were created as single-choice questions, multiple-choice questions, or free-response questions. A team reviewed the survey and applied minor changes to enhance usability and readability (see questionnaire in German as Supplementary Information). The survey was anonymous and voluntary. For the distribution of the questionnaire, we used the online platform survio.com. The platform ensured data protection and security (2048-bit SSL security, ISO/IEC 270001 standards, daily backups). We sent a hyperlink via e‑mail to registered members of the German Society of Radiation Oncology (DEGRO) as well as to members of the professional associations of medical physics experts, radiology assistants/radiation therapists, nurses, and administrative personnel in radiation oncology. The survey was available for completion between May 23, 2020 and June 9, 2020. All statistical analysis was performed using SPSS version 25 (IBM, Armonk, NY, USA).

## Results

In total, 334 participants completed the survey between May 23, 2020 and June 9, 2020. Table 1 shows the participant characteristics.

**Table 1:** Participant characteristics (*n* = 334)

	*n* (%)
*Gender*	
Female	236 (70.7)
Male	95 (28.4)
Other	3 (0.9)
*Institution*	
University hospital	137 (41.0)
Non-university hospital	47 (14.1)
Ambulatory health care centre	78 (23.4)
Medical practice	72 (21.5)
*Position*	
Physicians	120 (35.9)
Radiology assistant/Radiation therapist	84 (25.1)
Nurse/Medical assistant	34 (10.2)
Administrative personnel	25 (7.5)
Medical physics experts	68 (20.4)
Other	3 (0.9)
*Country*	
Germany	320 (95.8)
Austria	7 (2.1)
Swiss	7 (2.1)
Other	0 (0)
*Participants with children who need care*	
Yes	97 (29.0)
No	237 (71.0)
*SARS-CoV‑2 risk group*	
Yes	94 (28.1)
No	240 (71.9)

**Fig. 1:**
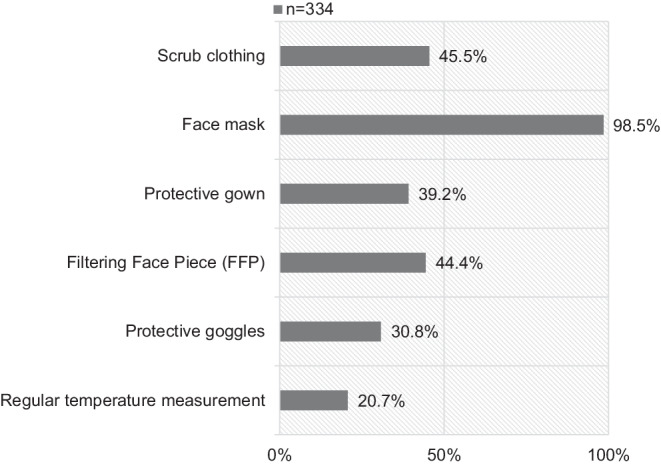
Protective measures during the SARS-CoV‑2 pandemic

Of the participants, 18.9% (63/334) stated that their institution had a pandemic plan, while 37.1% (124/334) declined having a plan (unknown: 44.0%, 147/334). 26.3% (88/334) and 44.6% (149/334) of the participants felt that their institution was fully or rather prepared for the pandemic (less prepared: 24.3%, 81/334; not prepared: 4.8%, 16/334). Fig. 1 shows the protective measures for HCPs during the pandemic.

In 66.2% (221/334) of the cases, HCPs reported about a shortage in protective clothing (no shortage: 33.8%, 113/334). Due to the shortage in protective clothing, 33.5% (112/334) stated that the clothing was reprocessed for reuse (no reuse: 66.5%, 222/334).

**Fig. 2:**
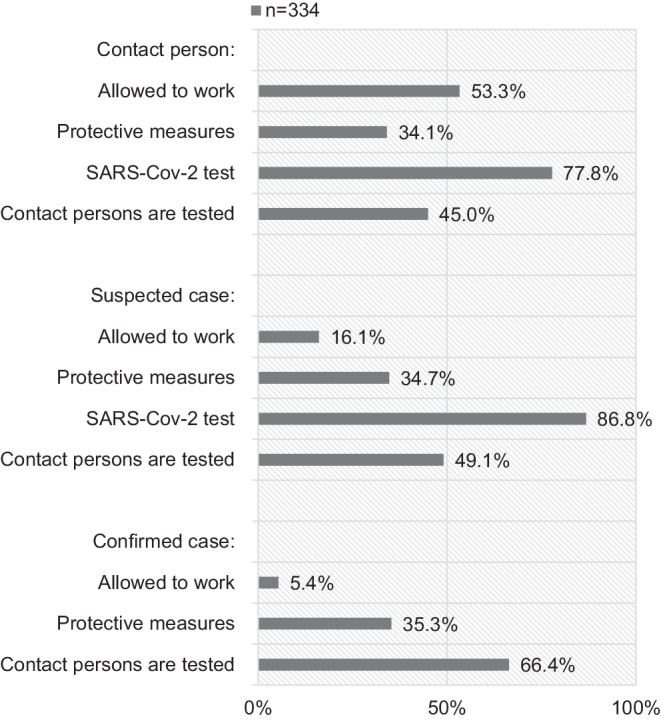
Measures for health care professionals identified as contact persons, suspected cases and confirmed cases regarding SARS-CoV‑2

Of the participants, 79.6% (266/334) stated that all patients in their institution received face masks, while 5.1% (17/334) of the participants reported that only SARS-CoV-2-positive patients received masks (no masks: 15.3% 51/334). Access restrictions were established in 96.1% (321/334) of the participants’ institutions (no access restrictions: 3.9%, 13/334). Fig. 2 shows the institutions’ measures for HCPs identified as contact persons, suspected cases and confirmed cases regarding SARS-CoV‑2.

Fig. 2 shows the measures for HCPs identified as contact persons (contact to a confirmed case < 14 days), suspected cases (persons with respiratory symptoms, fever and contact to a confirmed case), or confirmed cases (positive SARS-CoV‑2 test).

The protective measures were regarded as very reasonable by 47.4% (158/334), while 0.8% (3/334) regarded them as not reasonable (rather reasonable: 44.0%, 147/334; less reasonable 7.8%, 26/334). 32.9% (110/334) of the participants felt sufficiently protected (rather sufficiently protected: 48.5%, 162/334; less sufficiently protected: 16.2%, 54/334; not sufficiently protected: 2.4%, 8/334).

Of all, 28.1% (94/334) identify themselves as members of the SARS-CoV‑2 risk group due to age or medical history (Table 1). In 28.1% (94/334), the employers choose to release members of the SARS-CoV‑2 risk group from work (no release: 71.9%, 240/334). 36.5% (122/334) of the participants stated that antibody tests were used to select employees with a past infection for work with positive patients.

Participants treated an average number of 2 SARS-CoV-2-positive patients (range: 0–143). 2.9% (10/334) of the participants have tested positive themselves: five of them without symptoms. An average number of 0.8 individuals of family/friends tested positive during the pandemic (range: 0–30). Of the colleagues, an average of 1.2 (range: 0–40) was tested positive. In 67.4% (225/334) of the cases, no information on the nature of the infection existed. In 23.4% (78/334) the infection took place in the private area, while 9.2% (31/334) took place in the workplace. 6.6% (22/334) and 15.6% (52/334) were fully or rather scared to infect themselves, while 57.2% (191/334) and 20.6% (69/334) were less and not scared about transmission.

**Fig. 3:**
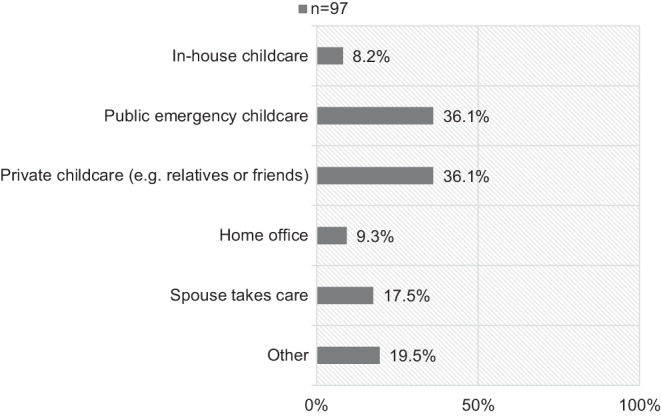
Childcare organized by the health care professionals with children during the SARS-CoV‑2 pandemic

Of the participants, 29.0% (97/334) had children who needed care (Table 1). Fig. 3 shows how those parents organized the childcare during the pandemic.

The SARS-CoV‑2 pandemic led to staff shortages in the roster in 24.9% (83/334) (no staff shortage: 75.1%, 251/334). The weekly overtime was 4.2 h on average (range: 0–385 h). 27.2% (91/334) and 34.4% (115/334) fully or rather agreed with the fact of additional work burden due to the pandemic (agreed less: 28.2%, 94/334; did not agree: 10.2%, 34/334).

Regarding less time available for their private life, 12.0% (40/334) and 23.9% (80/334) fully or rather agreed, while 40.4% (135/334) and 23.7% (79/334) agreed less or not at all. 11.7% (39/334) and 29.6% (99/334) fully and rather agreed with reduced work satisfaction due to the SARS-CoV‑2 pandemic (agreed less: 39.8%, 133/334; did not agree: 18.9%, 63/334). 23.4% (78/334) and 34.7% (116/334) fully and rather agreed with the fact that their employer appreciated their work during the pandemic; 31.4% (105/334) and 10.5% (35/334) agreed less or did not agree, respectively.

Furthermore, 12.9% (43/334) and 29.0% (97/334) of the participants were fully or rather mentally strained by the SARS-CoV‑2 pandemic (less mentally strained: 44.0%, 147/334; not mentally strained: 14.1%, 47/334). In 48.5% (162/334), participants stated that psychological counselling was offered in their institution.

Finally, 47.3% (158/334) of the participants use a mobile application for SARS-CoV‑2 warning.

## Discussion

The SARS-CoV‑2 pandemic has a significant impact on everyone’s private and work life. Especially in medical disciplines in which oncological patients are treated, the workflow has changed and comes with even greater responsibilities. We conducted a survey on the effects of the SARS-CoV‑2 pandemic on HCPs in radiation oncology. The data clearly show that the pandemic itself, as well as the new measures and requirements, the daily changing information and the increase of anxiety have an impact in several aspects of HCPs in radiation oncology.

The last big global pandemic in history was the Spanish flu in 1918 to 1920 with an estimated 50–100 million deaths [12]. Since then, our health care system has never been so severely challenged. Consequently, the question of whether or not our health care system was and is prepared for a global pandemic is essential. Only 26.3% and 44.6% of the participants felt that their institution was fully or rather prepared for the pandemic, with only 18.9% knowing of a pandemic plan. Paffenholz et al. evaluated answers of 2827 HCPs of all medical disciplines in Germany and showed that only 34.1% and 3.5% found that Germany was well and very well prepared for the pandemic. A 2012 risk analysis of the German Federal Office for Citizen Protection and Disaster Assistance (BKK) with the hypothetical virus “Modi-SARS” rated such a scenario as probable with a statistical probability of one event in 100 to 1000 years [13]. Further, a survey of the Bavarian Hospital Society in 2007 revealed that only 59% of the hospitals had pandemic plans in cases of an influenza outbreak [14]. However, most of the pandemic plans waited in the drawer until 2020 and even then, the execution of the plans was not entirely fluid. Such plans are essential for oncological disciplines such as radiation oncology, as oncologists deal with immunosuppressed cancer patients who might be more vulnerable to SARS-CoV‑2 than others.

A crucial part of the fight against the virus spread during the pandemic includes the protective measures. Nearly all participants (98.5%) reported the use of face masks in their institution, while filtering face pieces (FFP) masks were only used by 44.4%. In our survey, 66.2% of the HCPs reported about a shortage of protective clothing with a reuse of the clothing in 33.5%. The shortage of protective clothing has been reported before [15, 16]. Furthermore, this is in line with the survey of Paffenhof et al., in which 27.5% of the participants reported a short-term shortage, while over 40% confirmed a regular or permanent shortage of consumable goods [17].

Only about 80% of the participants stated that all patients received face masks in their institution during the pandemic. In recent times, Germany has introduced the face mask as one of the most critical measures in the fight against the virus spread. During the first wave, discussions about the benefit were held across the world. However, today we know that face masks have a protective effect [18].

The measures undertaken in case of contact (contact to a confirmed case < 14 days), suspected cases (persons with respiratory symptoms, fever and contact to a confirmed case) or confirmed cases (positive SARS-CoV‑2 test) are shown in Fig. 2. The definitions result from the rules of the Robert Koch Institute (RKI) valid at the time of the first pandemic wave [19, 20]. Contact persons as well as suspected cases were tested in large numbers. Contact persons were allowed to work in more than half of the cases. This might be owed to the fact that in early 2020 HCPs were kept on stand-by for the feared virus outbreak seen in Italy, Spain and the United States. However, quarantining is essential, since Hellewell et al. showed that isolation and contact tracing is enough to control new SARS-CoV‑2 outbreaks within three months in most scenarios [21]. Recently, contact persons are obliged to quarantine to interrupt possible infection chains which is essential to protect vulnerable patients, especially oncologic patients. 28.1% of the participating HCPs who identified themselves as members of the SARS-CoV‑2 risk group also stated that the employers choose to release members of the SARS-CoV‑2 risk group from work. We believe that it was partly common to release HCPs with risk factors in the eye of the upcoming first wave. However, as the survey focused on individual answers of HCPs and not on single institutions, the number in our evaluation might be too high.

Overall, 91.4% of the participants found the protective measures fully or rather reasonable, and 81.4% felt sufficiently or rather sufficiently protected by them. Although there seems to be a broad consensus, it should be noted that the protective measures differed between institutions (Fig. 1). Paffenholz et al. reported that 40.3% and 15.3% rated their employer’s measures against SARS-CoV‑2 as positive and very positive [17]. The higher rate of agreement in our study might be owed to the timing of our survey, which took place in May and June compared to the survey of Paffenholz et al. which was conducted earlier in March and April [17].

Many participants in our survey mention overtime, additional work burden, and less time for their private life. With the pandemic and the lockdown, the daily routine of all HCPs changed. Paffenholz et al. showed that the daily work routine has changed strongly (41.9%) or very strongly (40.0%) for German HCPs [17]. Further, the authors showed that most participants are very strongly (30.7%) or strongly (44.7%) affected in their private life [17]. Compared to our data, it seems like HCPs in radiation oncology are less affected by the current situation as their colleagues from throughout Germany. This might be owed to the fact that HCPs in radiation oncology are not directly involved in the treatment of SARS-CoV‑2 positive patients which was the main focus of the other surveys. Consequently, only 6.6% and 15.6% of the participants in our survey were fully or rather scared to infect themselves. Furthermore, the number of positive patients was lower in the first wave of the pandemic than nowadays in view of the omicron wave. Therefore, the possibility of coming into contact with positive patients was lower in radiation oncology departments than in internal medicine or surgery. In our survey, the participants only treated an average of 2 SARS-CoV-2-positive patients (range: 0–143).

Given the pandemic and the lockdown in many countries, the systemic importance of HCPs is beyond doubt. Therefore, childcare for such systemically relevant groups is essential. 29.0% of our participants had children who needed care. Most of the participants used the public emergency childcare or private options (e.g. relatives or friends). Stress and anxiety for HCP parents are probably higher as for HCPs without children due to the worries of the health status of their family and care options during the pandemic. Fong et al. [22] applied a systemic review on previous data on mental health outcomes during social isolation and quarantining for parents and children and drew implications for the present SARS-CoV‑2 pandemic. They showed that parents experience high stress and anxiety during pandemics, especially for HCPs. Therefore, Fong et al. advocate for better protection.

In our survey, 12.9% and 29.0% of the participants were fully or rather mentally strained by the SARS-CoV‑2 pandemic. Recently, Vizheh et al. showed that HCPs face psychological pressure and even mental illness during the pandemic [23]. Thomaier et al. found that the SARS-CoV‑2 pandemic and the resulting interference with cancer care was associated with anxiety and depression symptoms among cancer physicians in the United States [24]. However, only half of the participants in our cohort stated that psychological counselling was offered in their institution. Studies have suggested a positive impact of such interventions during the SARS-CoV‑2 pandemic [25–27]. However, Pollock et al. found a lack of evidence from studies during or after disease pandemics that suggest the selection of specific interventions [28]. Therefore, future research should aim at evaluating such interventions.

Overall, 41.3% of the participants agreed with reduced work satisfaction due to the pandemic. Further, 41.9% of the HCPs felt that their work during the pandemic was not appreciated enough. This is crucial for the mental health of the front-line workers as well as for the work performance. Travers et al. evaluated the influence of empowered work environments on nursing assistants during the pandemic and showed that empowerment of HCPs is vital to hospital performance and success [29].

Putting everything in a nutshell, what can we as radiation oncology departments learn from the pandemic for future similar situations? Protective clothing must be sufficiently available. This is important for the protection of all front-line workers and patients, but even more so in radiation oncology to protect vulnerable oncological patients. Therefore, politics and economy should not only rely on the production of such protective clothing in foreign countries. At the time of the publication of this article, protective clothing is widely available in Germany. However, for future similar situations, the sufficient provision of such clothing must be possible in a shorter time. This should be an integral part of the pandemic management.

Further, it is crucial to break infection chains consequently. Positive HCPs and contact persons should be quarantined if the workload allows for that. That way, the risk for a virus outbreak among the staff and transmission to patients might be reduced. Nowadays, this is an important part of the pandemic management. In contrary to the first pandemic wave, a vaccination is available. Since vaccinated persons do not have to quarantine as contact persons, this reduces the number of HCPs not able to work. However, although Holzmann-Littig et al. [30] observed a high acceptance concerning vaccination among German HCPs (91.7%), a few HCPs are still hesitant. Therefore, quarantining for HCPs is also essential in the future.

Childcare must be ensured and considered in pandemic plans—either with public emergency childcare or programs by the department itself. Thus, anxiety and stress for HCP parents might be reduced so that they can focus on their work. Furthermore, mental health support during a pandemic is crucial. Working with cancer patients is emotionally and mentally demanding, per se. Therefore, psychological counselling should be offered to all HCPs in radiation oncology during this time. Moreover, work satisfaction and appreciation by employers is essential. Different approaches to enhance that (e.g. financial appreciation, free food in lunchtimes) should be evaluated.

All of these measures should be considered for future similar situations. Postponing treatment of cancer patients is not an option in a pandemic, so establishing standard operating procedures for pandemic situations is of utmost importance, especially for oncological departments like radiation oncology. This study may only highlight more trivial aspects of medical care, but overall they have immense implications for the best possible treatment of cancer.

Our study has certain limitations which are inherent to online questionnaires. We could not control for how many participants of one single institution participated in the survey. Therefore, the practice of institutions with a higher number of participants might also have a greater influence on the results. However, the goal of this survey was to evaluate the situation of individual HCPs. Further, we could not calculate a response rate due to the online format. However, a very large group of professionals took part in the survey which also reflects the interest and concern about all factors associated with the SARS-CoV‑2 pandemic. Based on this large group, the data provide very good evidence on the factors that worry HCPs and must be focused on clearly in the future.

## Conclusion

The severe acute respiratory syndrome coronavirus 2 (SARS-CoV-2) pandemic had an impact on the daily work and private life of health care professionals (HCPs) in radiation oncology units. We learned that employers should ensure availability of protective clothing and consequent tracing of infection chains among the HCPs. Measures like childcare programs are essential and remain a major obstacle. A very central aspect is that mental health support is crucial and should be provided considering that HCPs working in the field of oncology are exposed to demanding and emotionally challenging work. Furthermore, work satisfaction and appreciation of HCPs and their work by employers is essential and contributes significantly to emotional well-being of the workforce.

## Supplementary Information

Questionnaire (in German)
