# Supplementary material for: Coronavirus disease 2019 and radiation oncology—survey on the impact of the severe acute respiratory syndrome coronavirus 2 pandemic on health care professionals in radiation oncology
Source: Strahlenther Onkol. 2022 Feb 23;198(4):346–53. doi: 10.1007/s00066-022-01903-8 (PMC8864974; doi:10.1007/s00066-022-01903-8)
Supplement: Supplementary file 1 — Questionnaire (in German) [file 66_2022_1903_MOESM1_ESM.pdf]

Umfrage zu Auswirkungen der SARS-CoV-2-Pandemie auf die Mitarbeiter*innen in der Radioonkologie

Sehr geehrte*r Kollegen*innen,

herzlichen Dank für die Teilnahme an unserer Umfrage zu den Auswirkungen der SARS-CoV-2-Pandemie auf die Mitarbeiter*innen in der Radioonkologie. Sie wird ca. 10 Minuten Ihrer Zeit beanspruchen.

Bei Rückfragen stehen wir Ihnen natürlich immer gerne zur Verfügung.

Beste Grüße aus München

Ihre

Univ.-Prof. Dr. Stephanie E. Combs

Direktorin der Klinik und Poliklinik für RadioOnkologie und Strahlentherapie

Klinikum rechts der Isar, Technische Universität München (TUM)

In welchem Land sind Sie tätig?

- ◯ Deutschland
- ◯ Österreich
- ◯ Schweiz
- ◯ Andere ______

Welches Geschlecht haben Sie?

- ◯ weiblich
- ◯ männlich
- ◯ divers*

In welcher Einrichtung sind Sie tätig?

- ◯ Einzel- oder Gemeinschaftspraxis
- ◯ Medizinisches Versorgungszentrum (MVZ)/ Primärversorgungszentrum (PVZ)
- ◯ Nicht-universitäre Klinik/Spital
- ◯ Universitätsklinik/-spital

In welcher Position sind Sie tätig?

- ◯ Arzt*Ärztin in Weiterbildung
- ◯ Angestellter*e Facharzt*ärztin
- ◯ Inhaber*in
- ◯ Medizinisch-technische*r Radiologieassistent*in
- ◯ Medizinphysik-Experte*in
- ◯ Pflegepersonal
- ◯ Medizinische*r Fachangestellte*r
- ◯ Verwaltungspersonal
- ◯ Andere ______________________

In welcher Position sind Sie tätig?

- ◯ Assistenzarzt*ärztin
- ◯ Facharzt*ärztin
- ◯ Oberarzt*ärztin
- ◯ Chefarzt*ärztin
- ◯ Medizinisch-technische*r Radiologieassistent*in
- ◯ Medizinphysik-Experte*in
- ◯ Pflegepersonal
- ◯ Medizinische*r Fachangestellte*r
- ◯ Verwaltungspersonal
- ◯ Andere ______________________

Hatten Sie in Ihrer Einrichtung vor Beginn der SARS-CoV-2-Pandemie einen Pandemieplan?

- ◯ Ja
- ◯ Nein
- ◯ Ist mir nicht bekannt

Welche Schutzmaßnahmen waren/sind durch Ihre*n Arbeitgeber*in für Sie bzw. Ihre Berufsgruppe während der SARS-CoV-2-Pandemie vorgesehen? (Mehrfachauswahl möglich)

- ☐ Bereichskleidung
- ☐ Mund-und-Nasen-Schutz (MNS)
- ☐ Schutzkittel
- ☐ Filtrierende Halbmasken (FFP)
- ☐ Schutzbrille/-visier
- ☐ Regelmäßige Temperaturmessungen

Erfolgte/Erfolgt in Ihrer Einrichtung die Ausstattung von Patienten*innen mit Mund-Nasen-Schutz (MNS)?

- ◯ Ja, für alle Patienten*innen
- ◯ Ja, für positiv getestete Patienten*innen
- ◯ Nein

Kam/Kommt es zu Engpässen für Schutzkleidung und Desinfektionsmittel im Rahmen der SARS-CoV-2-Pandemie?

- ◯ Ja
- ◯ Nein

Erfolgte/Erfolgt in Ihrer Einrichtung die Aufbereitung und Wiederverwendung von Schutzkleidung?

- ◯ Ja
- ◯ Nein

Fühlten/Fühlen Sie sich durch die Schutzmaßnahmen in Ihrer Einrichtung ausreichend geschützt?

- ◯ Trifft voll zu
- ◯ Trifft eher zu
- ◯ Trifft weniger zu
- ◯ Trifft gar nicht zu

Fühlten/Fühlen Sie sich durch Ihre*n Arbeitgeber*in ausreichend über SARS-CoV-2 informiert?

- ◯ Trifft voll zu
- ◯ Trifft eher zu
- ◯ Trifft weniger zu
- ◯ Trifft gar nicht zu

Wie wurde/wird in Ihrer Einrichtung mit Mitarbeitern*innen umgegangen, die als Kontaktpersonen (Kontakt <14 Tage zu bestätigtem Fall) galten/gelten?

	Ja	Nein
Darf arbeiten	◯	◯
Benötigt spezielle Schutzkleidung	◯	◯
Wird auf SARS-CoV-2 getestet	◯	◯
Kontaktpersonen werden getestet	◯	◯

Wie wurde/wird in Ihrer Einrichtung mit Mitarbeitern*innen umgegangen, die als Verdachtsfälle (Personen mit akuten respiratorischen Symptomen jeder Schwere, ggf. Fieber UND Kontakt mit einem bestätigten Fall von SARS-CoV-2) gelten/galten?

	Ja	Nein
Darf arbeiten	◯	◯
Benötigt spezielle Schutzkleidung	◯	◯
Wird auf SARS-CoV-2 getestet	◯	◯
Kontaktpersonen werden getestet	◯	◯

Wie wurde/wird in Ihrer Einrichtung mit Mitarbeitern*innen umgegangen, die positiv auf SARS-CoV-2 getestet wurden/werden?

	Ja	Nein
Darf arbeiten	◯	◯
Benötigt spezielle Schutzkleidung	◯	◯
Kontaktpersonen werden getestet	◯	◯

Gehören Sie zur SARS-CoV-2-Risikogruppe (z. B. aufgrund von Vorerkrankung oder Alter)?

◯ Ja

◯ Nein

Erfolgte/Erfolgt die Freistellung von Mitarbeitern*innen mit Risikofaktoren?

◯ Ja

◯ Nein

Wie viele SARS-CoV-2 positive Patienten*innen haben Sie bisher behandelt oder mitbehandelt?

Hatten/Haben Sie Angst sich bei Ihrer Tätigkeit mit SARS-CoV-2 anzustecken?

- ◯ Trifft voll zu
- ◯ Trifft eher zu
- ◯ Trifft weniger zu
- ◯ Trifft gar nicht zu

Wurden Sie bereits positiv auf SARS-CoV-2 getestet?

- ◯ Ja
- ◯ Nein

Wenn ja, welche Symptome hatten Sie? (Mehrfachauswahl möglich)

- ☐ Keine Symptome
- ☐ Husten
- ☐ Fieber
- ☐ Atembeschwerden
- ☐ Gliederschmerzen
- ☐ Geruchs-/Geschmacksstörungen
- ☐ Andere

Wenn ja, erfolgte die Testung über Ihre*n Arbeitgeber*in?

- ◯ Ja
- ◯ Nein

Wie viele Freunde/Verwandte/Angehörige in Ihrem näheren Umfeld wurden bereits positiv auf SARS-CoV-2 getestet?

Wurde/Wird in Ihrer Einrichtung eine Zutrittsbeschränkung für Angehörige verhängt?

- ◯ Ja
- ◯ Nein

Halten Sie die ergriffenen Schutzmaßnahmen für angemessen?

- ◯ Trifft voll zu
- ◯ Trifft eher zu
- ◯ Trifft weniger zu
- ◯ Trifft gar nicht zu

War/Ist Ihre Einrichtung Ihrer Meinung nach auf die SARS-CoV-2-Pandemie gut vorbereitet?

- ◯ Trifft voll zu
- ◯ Trifft eher zu
- ◯ Trifft weniger zu
- ◯ Trifft gar nicht zu

Wie viele Mitarbeiter*innen in Ihrer Einrichtung sind an SARS-CoV-2 bis zum jetzigen Zeitpunkt erkrankt?

	Erkrankte Mitarbeiter*innen	Gesamtanzahl Mitarbeiter*innen
Anzahl		

Gibt es Erkenntnisse darüber, wo sich diese Mitarbeiter*innen überwiegend angesteckt haben?

- ◯ Darüber gibt es keine Erkenntnisse
- ◯ Überwiegend im Rahmen der Tätigkeit in der Einrichtung
- ◯ Überwiegend im privaten Bereich

Kam/Kommt es aufgrund der Anzahl von SARS-CoV-2-erkrankten Mitarbeiter*innen zu Engpässen bei der Dienstplangestaltung oder Mehrbelastung?

- ◯ Ja
- ◯ Nein

Wurden/Werden in Ihrer Einrichtung Antikörpertests durchgeführt, um eine überstandene Infektion zu erkennen und so Mitarbeiter*innen gezielt einsetzen zu können?

- ◯ Ja
- ◯ Nein

Hat die SARS-CoV-2-Pandemie zu einer Steigerung Ihrer Arbeitsbelastung geführt?

- ◯ Trifft voll zu
- ◯ Trifft eher zu
- ◯ Trifft weniger zu
- ◯ Trifft gar nicht zu

Hat sich seit Beginn der SARS-CoV-2-Pandemie Ihre Arbeitszufriedenheit verschlechtert?

- ◯ Trifft voll zu
- ◯ Trifft eher zu
- ◯ Trifft weniger zu
- ◯ Trifft gar nicht zu

Haben Sie betreuungspflichtige Kinder?

- ◯ Ja
- ◯ Nein

Welche Betreuungsmöglichkeiten nutzten/nutzen Sie während der SARS-CoV-2-Pandemie? (Mehrfachauswahl möglich)

- ☐ Ich nutzte/nutze eine Einrichtungs-interne Kinderbetreuung
- ☐ Ich nutzte/nutze eine Notfallbetreuung in öffentlichen Einrichtungen
- ☐ Ich nutzte/nutze eine private Betreuungsmöglichkeit
- ☐ Ich arbeite im Home Office und betreute/betreue meine Kinder selbst
- ☐ Andere __________

Führte/Führt die Betreuungssituation Ihrer Kinder während der SARS-CoV-2-Pandemie zu einer Mehrbelastung?

- ○ Trifft voll zu
- ○ Trifft eher zu
- ○ Trifft weniger zu
- ○ Trifft gar nicht zu

Haben Sie durch die SARS-CoV-2-Pandemie deutlich weniger Zeit für Ihr Privatleben?

- ○ Trifft voll zu
- ○ Trifft eher zu
- ○ Trifft weniger zu
- ○ Trifft gar nicht zu

Haben Sie das Gefühl, dass Ihre Arbeit während der SARS-CoV-2-Pandemie von Ihrer*m Arbeitgeber*in wertgeschätzt wird?

- ○ Trifft voll zu
- ○ Trifft eher zu
- ○ Trifft weniger zu
- ○ Trifft gar nicht zu

Wie viele Überstunden machen Sie im Durchschnitt zusätzlich seit Beginn der SARS-CoV-2-Pandemie pro Woche?

Fühlen Sie sich durch die SARS-CoV-2-Pandemie psychisch belastet?

- ◯ Trifft voll zu
- ◯ Trifft eher zu
- ◯ Trifft weniger zu
- ◯ Trifft gar nicht zu

Gibt es in Ihrer Einrichtung die Möglichkeit einer psychologischen Betreuung im Rahmen der SARS-CoV-2-Pandemie?

- ◯ Ja
- ◯ Nein

Benutzen Sie die Cornona-Warn-App des Robert-Koch-Instituts oder eine andere Corona-App?

- ◯ Ja
- ◯ Nein

Haben Sie Kommentare oder Anmerkungen?

Vielen Dank, dass Sie sich die Zeit genommen haben an der Umfrage teilzunehmen!

Beste Grüße aus München

Ihre

Univ.-Prof. Dr. Stephanie E. Combs
